# Modelling the bioinformatics tertiary analysis research process

**DOI:** 10.1186/s12859-021-04310-5

**Published:** 2021-09-30

**Authors:** Sara Pidò, Pietro Crovari, Franca Garzotto

**Affiliations:** Department of Electronics, Information and Bioengineering, Politecnico di Milano, Milan, Italy

**Keywords:** Bioinformatics, Tertiary analysis, Hierarchical task tree, Research methodology, User study

## Abstract

**Background:**

With the advancements of Next Generation Techniques, a tremendous amount of genomic information has been made available to be analyzed by means of computational methods. Bioinformatics Tertiary Analysis is a complex multidisciplinary process that represents the final step of the whole bioinformatics analysis pipeline. Despite the popularity of the subject, the Bioinformatics Tertiary Analysis process has not yet been specified in a systematic way. The lack of a reference model results into a plethora of technological tools that are designed mostly on the data and not on the human process involved in Tertiary Analysis, making such systems difficult to use and to integrate.

**Methods:**

To address this problem, we propose a conceptual model that captures the salient characteristics of the research methods and human tasks involved in Bioinformatics Tertiary Analysis. The model is grounded on a user study that involved bioinformatics specialists for the elicitation of a hierarchical task tree representing the Tertiary Analysis process. The outcome was refined and validated using the results of a vast survey of the literature reporting examples of Bioinformatics Tertiary Analysis activities.

**Results:**

The final hierarchical task tree was then converted into an ontological representation using an ontology standard formalism. The results of our research provides a reference process model for Tertiary Analysis that can be used both to analyze and to compare existing tools, or to design new tools.

**Conclusions:**

To highlight the potential of our approach and to exemplify its concrete applications, we describe a new bioinformatics tool and how the proposed process model informed its design.

**Supplementary Information:**

The online version contains supplementary material available at 10.1186/s12859-021-04310-5.

## Background

### Introduction

Bioinformatics Tertiary Analysis is defined as the use of complex computer science methods, algorithms and tools to understand and analyze the sequencing results extracted from raw genomic data [1, 2]. Tertiary Analysis activities represent the last mile of the bioinformatics pipeline that begins with the identification of raw data and the generation of sequencing reads (primary analysis) and their alignment (secondary analysis) [2, 3].

Since the introduction of Next Generation Sequencing techniques [4], bioinformatics Tertiary Analysis experienced a rapid growth given the increasingly large availability of genetic material to analyze. As a consequence, many tools have been developed to support researchers in this process.

As Bolchini stated in [5], the usability of bioinformatics tools is a severe problem that weakens their power to support bioinformatics research and their potential for adoption. Even if these applications were developed to support the bioinformatics activities and mitigate the difficulties that are intrinsic in the subject, many of these tools are perceived as very complex to learn and to use. If the user is not an expert in both biology and computer science, managing them requires a significant cognitive effort which should instead be devoted to answer research questions. This problem is more accentuated when biological data and operations increase in complexity. They require advanced algorithms and computer science methods that are often understood only by machine learning engines or data mining experts.

To the best of our knowledge, most bioinformatics applications were developed using a “system-centric” approach, i.e., focusing more on technological requirements than on the user needs and the characteristics of the human processes involved. To make these applications more usable and more useful, a more “user-centric” approach could be needed to take into consideration the bioinformatics researcher’s perspective since the very beginning of the technology design process. In particular, tools dedicated to bioinformatics research should require more concern about (i) the characteristics of the pipeline that is going to be used, (ii) the inputs available to the users, (iii) the preferred/required outputs, (iv) the existing relations among the process’ elements.

To address the above issues, our approach employs process models that are progressively refined to represent and make clear all the steps that are commonly taken by a bioinformatician in a typical Tertiary Analysis activity.

To elicit our process models, we first performed an exploratory user study involving eight bioinformaticians; they identified the tasks involved in Tertiary Analysis, conceptualized the process, and represented it as a hierarchical task tree using a well-known method called *hierarchical task analysis* [6] agreed among all participants. Then we refined and validated the process model using the outcomes of a survey of the literature reporting examples of bioinformatics Tertiary Analysis. At the end, we translated the final hierarchical task tree into an ontology-based representation using OWL—a typical formalism for ontologies.

Our main contributions are:

1. A tree-like conceptual representation of the steps of a Tertiary Analysis that is: (i) elicited from an initial set of domain experts; (ii) validated by means of a vast review of the literature; (iii) supported by a set of examples that show its completeness.
2. A rigorous ontological representation of the resulting process specifications; such ontology, described in a standard notation, provides a *reference process model* for bioinformatics Tertiary Analysis that can be used both to analyze and to compare existing tools, or to design new tools for bioinformaticians
3. An approach to elicit and model bioinformatics processes that could be generalized to scopes beyond Tertiary Analysis: it could be applied for technology design in many contexts, particularly those involving cognitively complex cognitive activities that need to be precisely defined starting from the discovery of non-explicit expertise of the main actors.

This paper improves and extends the work reported in [7]. With respect to the previous publication, here we present a novel validation of the user study results, mapping more than 35 research works found in the literature on the elicited process model, and describes the translation of the Hierarchical Task Tree into an ontology-based formalism.

### Tools for bioinformatics

Tertiary bioinformatics analysis is probably the most challenging phase in the whole bioinformatics pipeline, it consists of defining and implementing machine learning, data mining and statistical algorithms to inspect, examine, and interpret sequencing results [3].

The first bioinformatics tools for tertiary analysis were scripts or programs executable through the command line. As soon as research interest grew on the topic, tools with a Graphical User Interface (GUI) started to be developed. GUI-based tools can be divided into two main categories: tools to perform a specific operation and tools that support the creation of research pipelines. Some examples of the first category are BEDTools [8], Bioconductor [9], Integrated Genome Browser [10]. Whereas, in the second family, the most famous are OrangeBioLab [11], UCSC Xena [12], Globus Genomics [13], and GenePattern [14].

BEDTools [8] is a toolset for genome arithmetic, i.e., set theory on the genome.

Bioconductor [9] uses the R statistical programming language to provide tools for the analysis and comprehension of high-throughput genomic data.

Integrated Genome Browser [10] is a visualization tool to explore and visually analyze biologically-interesting patterns in genomic datasets.

OrangeBioLab [11] is a visual tool for data visualization and analysis. Once the data have been uploaded, users can compose their workflow through a block interface. The platform provides modules for data mining, machine learning, feature scoring, predictive modeling, and data visualization.

UCSC Xena [12] interface allows researchers to visualize and compare data along multiple dimensions. Users can add or remove visualizations and interact with them. The columns-based layout allows us to see at a glance how the observed dimensions change between different samples.

Globus Genomics [13] environment has been developed to create graphical workflows for the analysis of genomic data. It provides tools for both data analysis and management, focusing on the possibility of sharing works among collaborators. Globus’s visual programming environment is based on Galaxy framework [15], a compelling environment for bioinformatics, but with a major focus on secondary analysis.

GenePattern [14] is a modular system that provides hundreds of genomic analysis tools through a visual interface. Its modules can be accessed through a block-based environment via browser or executed through code, inside Python Notebooks, or via command line. Everything in the system is highly customizable to adapt the tools to the specific problem.

New advancements in conversational technologies brought to the development of new dialogue-based interfaces for data retrieval, exploration, and analysis in recent years. These new tools exploit the power of Natural Language Understanding and Artificial Intelligence algorithms to create interfaces that minimize the learning barrier. Users can interact with written conversational agents (i.e., chatbots) to express the operation using natural language. The interfaces guide them through the process and transform users’ utterances into operations to be performed on data. Some examples are Iris [16] and Ava [17] for general data science, Maggie [18], BioGraphBot [19], and Ok DNA! [20] in the bioinformatics domain.

Ava [17] chatbot guides the user through a predefined analysis pipeline. Through the conversation, the method and parameters can be selected. Users do not need to know programming languages since the conversation produces executable code. The operations sequence, though, is fixed. Users can not modify it. The output of the process is a Python Notebook that can be executed to reproduce the experiment without the need to repeat the conversation.

Iris [16], instead, leaves users free to compose operations as preferred. Conversational units act as a wrapper for python functions that can be nested as desired, as long as the composition’s syntax is correct. Also in this system, the dialogue is converted into an executable Python Notebook, built step by step while the conversation evolves.

Maggie [18] focuses on bioinformatics data retrieval. In fact, it allows extracting data from BioCatalog through a conversational interface. The user, though, is not actively supported during the process by the conversational agent.

BioGraphBot [19], instead, translates users’ utterances into Gremlin Queries, to extract data from BioGraphDB. In this case, the user must know the structure of the underlying database to be able to use the chatbot.

Ok DNA! [20] actively supports users in data retrieval from genomic databases, removing the requirements of knowing the database structure. Users are actively guided until the query is complete so that also biologists and clinicians can use it even without a great computer science expertise.

### Elicitation and modeling of tasks requirements

The design of useful and efficient systems requires an in-depth knowledge of the user tasks that must be supported, as observed in a dated—but still very relevant—reference [21]. For this reason, before designing a new platform, it is essential to elicit all the tasks that the user will perform. Gaining such knowledge is a process called Task Model Elicitation in Human–Computer Interaction [22].

Over the years, many methodological frameworks have been produced with this aim. One of the most famous and adopted is GOMS framework [23]. GOMS states four fundamental elements at the base of every task, i.e., *Goals, Operators, Methods,* and *Section Rules*. According to the model, each task can be described according to a composition of those elements. Indeed, tasks are not atomic but are built from sub-tasks that is in turn derived from other smaller sub-tasks. This results in a hierarchy or elements that is intrinsically represented as a tree.

This tree-like representation is advantageous for its simplicity and because it allows us to make the comparison among different models elicited for the same task. Since a tree is made of nodes and edges and has a well-defined structure, the comparisons among the different sub-tasks, their type, or the number of steps required to reach a leaf node come naturally.

GOMS has been declined in many variants through the years. Other widely used frameworks are MECANO [24], MOBI-B [25], TRIDENT [26] and TADEUS [27]. However, these formulations do not model the user knowledge as part of the task [22].

Another popular tree-based framework is ConcurTaskTree [28]. It considers not only the structural relationships among tasks (i.e., *part of* relationship), but also their temporal relationships.

Using ontologies to describe phenomena is a widely-adopted practice in computer science. An ontology is a formal description of a set of concepts within a domain and the relationships between them. Ontologies have been developed to describe many domains; they have been used as a mechanism to provide applications with domain knowledge and to facilitate the sharing of information [29], that are exploited particularly in biology and bioinformatics domains [30]. Within the bioinformatics community, the relevance of ontologies has been recognized, and work has begun on designing and sharing biomolecular ontologies [31].

BioPortal is the biggest portal that collects ontologies in Biomedical and Bioinformatics domains. Among those, the Ontology for Biomedical Investigations (OBI) [32] models the research processes from the samples’ acquisition to their processing and transformation into genomic data. OBI has been developed to provide a common lexicon to describe the research process and support interoperability between different data sources. It is based on OWL2 language specification, and it comprises more than 3,600 classes put in relation through more than 100 properties. At an upper level, it is composed by four types of classes, *processes, material entity, and role and processes*, inherited by the Basic Formal Ontology (BFO) [33], and *information content entities* from the Information Artifact Ontology (IAO) [34].

To the best of our knowledge, there have been no studies centered on eliciting the full bioinformatics tertiary analysis process, even if many researchers recognized the importance of basing the design of bioinformatics tools on models such as ontologies of the method [35, 36].

## Methods

**Fig. 1:**
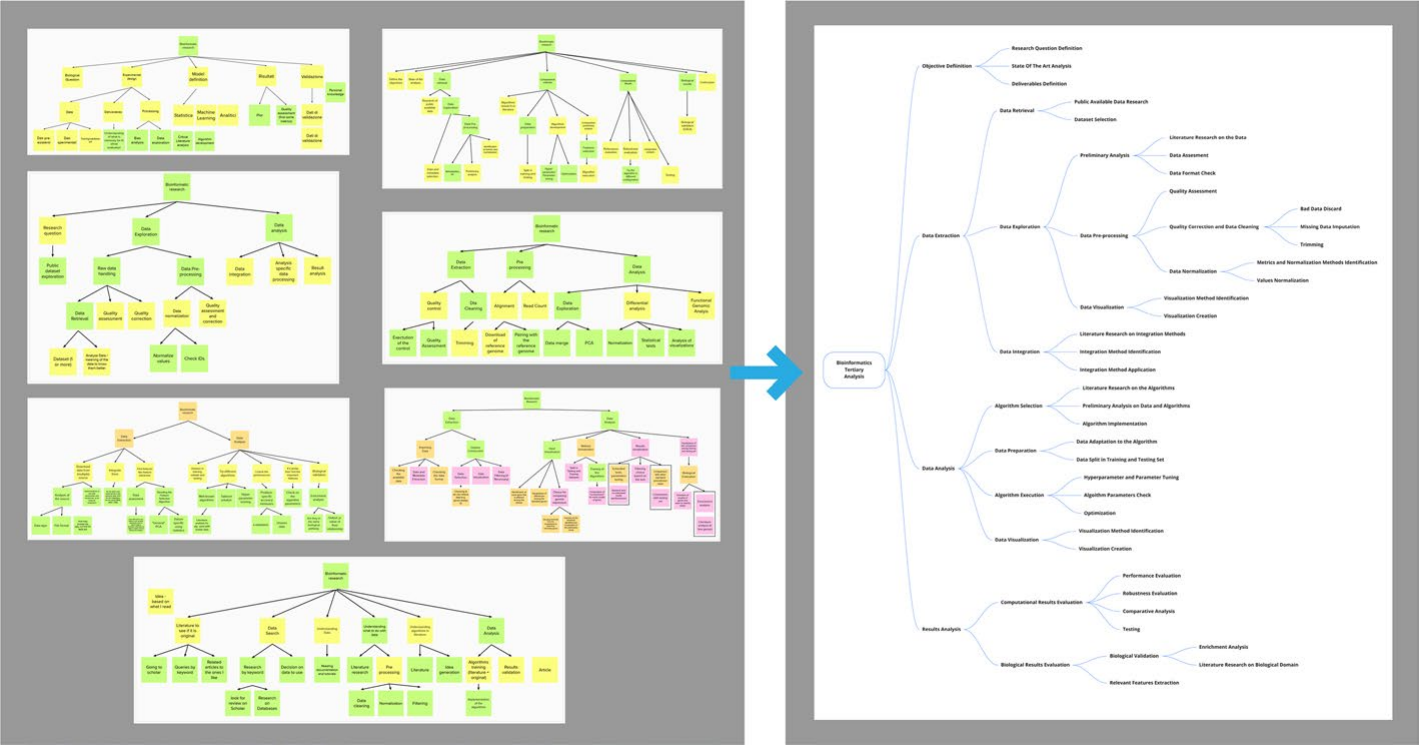
The three phases of users’ interviews. First, they had to describe their research process as they wanted. Then, we asked them to classify the steps according to their abstraction level by moving the sticky note accordingly. Finally, they had to complete the description of the tree adding the missing nodes

We describe how we generated a tree-based model of Bioinformatics Tertiary Analysis process starting from the users’ interviews, as depicted in Fig. 1. We started from eight semi-structured interviews with skilled bioinformaticians, asking them to describe their vision of the bioinformatics research process. Starting from their experience, each person interviewed defined a typical flow of their research activity, to build a hierarchical task tree, representing their own idea of bioinformatics tertiary research process. Subsequently, we combined these trees to generate a single one that is our starting model for the definition of a general bioinformatics tertiary analysis. Starting from the interviews’ results, the hierarchical task tree was the best method we found to illustrate the bioinformatics research process. Finally, we refined and validated it through the analysis of literature works, to assess its descriptive capabilities.

**Fig. 2:**
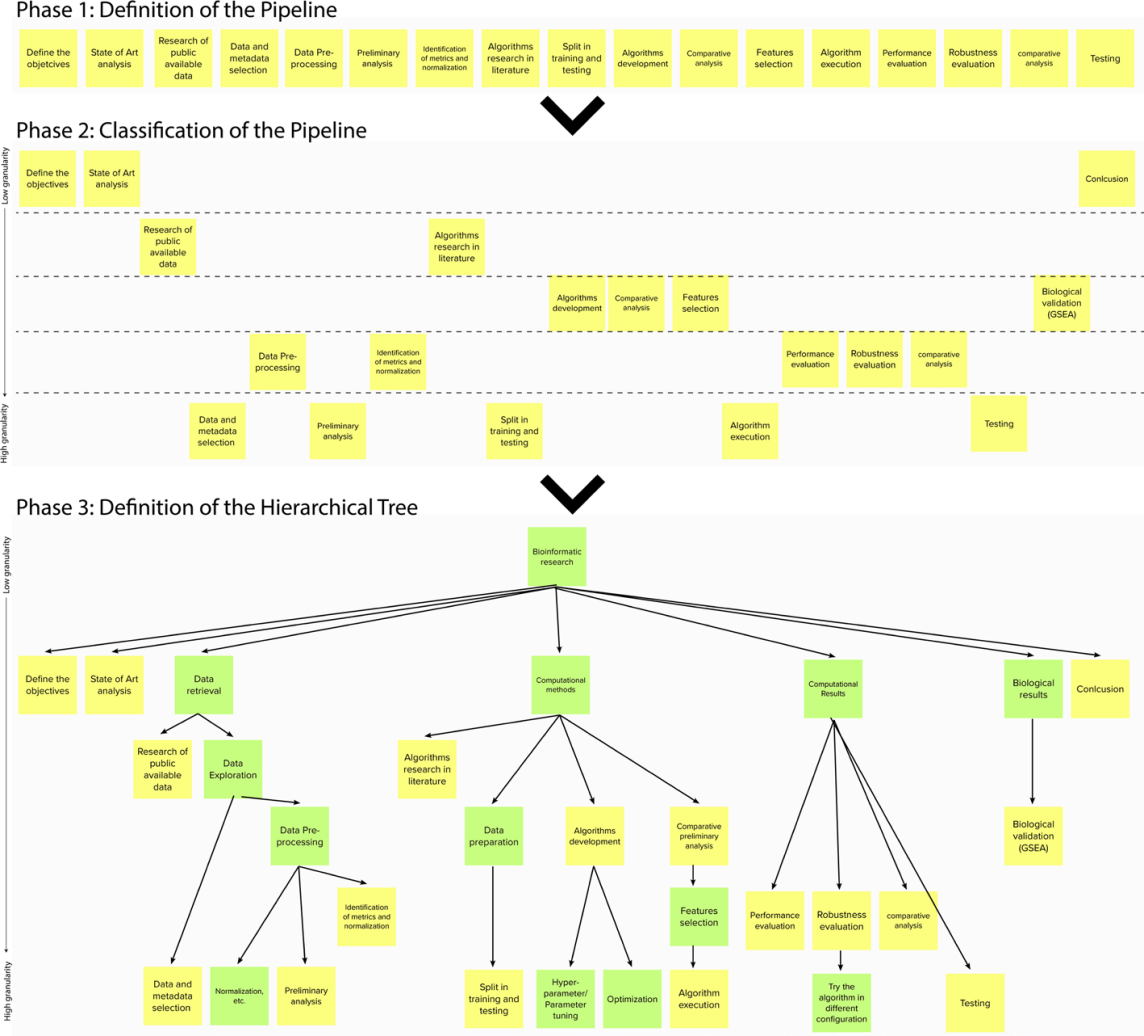
Methodology used for creating the model of the bioinformatics tertiary analysis process. We interviewed 8 bioinformatics experts. Each interview produced a hierarchical task tree. These were then merged in a single model. We validated the tree trying to map published research work on it. Finally, we modelled the ontology on the base of the tree

### User study

*Population.* To perform the study, we recruited eight expert bioinformaticians on a volunteer basis. Participants had heterogeneous academic roles: three Ph.D. students, two research assistants, two postdoctoral researchers, and one assistant professor.

*Setting.* Due to the current pandemic emergency outbreak, the user study was conducted in two different settings. During the first interviews, both the bioinformatician and the interviewer were in a room equipped with a table and a big whiteboard with sticky notes. With the advent of the lockdown, the interviews were carried out online during the quarantine through video conferencing software using the same protocol.

*Protocol.* Each individual interview was split into three main parts, as shown in Fig. 2. We provided the volunteer a whiteboard, either physical or virtual, both to help the interviewed person and to help us with a visual perspective. At the end of each phase we took a picture of the board, to be able to reconstruct the interview process during the results analysis. All the participants signed a consent form in which the study was explained in detail, including the guaranty on the anonymity of data collected.

The interview started with the *Definition of the pipeline*. We asked the participants to describe their typical research process step by step and invited them to use a sticky note for each step. No other constraints were given neither on the granularity of the steps nor on their number. We gave them as much freedom as possible during this first phase, and we interrupted their explanation only to ask for clarification.

The second phase was the *Classification of the pipeline*. Participants were required to classify their process elements according to the abstraction level. To do so, they use the board to organize their notes in layers according to their abstraction level. Since no granularity constraints on tasks was imposed during the prevous phase, the pipeline was always quite heterogeneous concerning the abstraction level of the description.

In the third and last phase, the *Definition of the Hierarchical-tree*, the participants were asked to build a hierarchical task tree of their typical research process: starting from the results of the first and the second phases of the interview, they connected one another to complete the whole tree.

*Results.* All the volunteers completed the elicitation of their hierarchical model successfully. The results of the first phases seemed very similar. Looking at the research flow and the larger granularity level, the comparison shows similar actions were considered in the same order even if the pipelines were different, and each one had its own abstraction level. Indeed, we were able to retrieve four typical macro-phases from all the processes immediately. These are also the typical phases of a data analysis pipeline: *Data Retrieval*, *Data Exploration*, *Data Analysis* and finally *Results Validation*. However, looking more carefully at the different results, we noticed that each participant focused on a different process step. This allowed us to retrieve a complementary perspective on the tertiary bioinformatics analysis and have a complete definition of each step.

We then evaluated the results of the second phase, i.e., the classification. Since each participant has its own abstraction level, the results were heterogeneous. Each participant had its own steps and sub-steps. However, we noticed that the interviewed attributed similar abstraction levels to similar operations. Almost every participant used three or four different abstraction levels in the classification process.

Finally, we studied the generated trees, reported in the Additional files 1–7. This analysis was divided into three main steps. The first one was the study of the topologies of the produced trees. Regarding the main backbone, it was similar among the whole interview set. However, particularly in the deepest nodes, the topologies of the trees were diverse. This because each participant’s focus was on a different workflow phase. Then, we compared the trees’ nodes and tried to produce a single tree with all the common nodes and the complementary ones. This comparison resulted in almost no conflicts. The few conflicts were in the leaf nodes of the tree. This allows us to point out that the researchers agree implicitly on how a tertiary bioinformatics analysis is usually carried out. As the last step, we accurately compared the produced tree with single ones. We analyzed the remaining nodes in the interviews’ trees. Even if they were few, we tried to adapt them to the new structure and, in case of compatibility, we added them.

### Hierarchical task tree

The described procedure leads to the creation of a description of the bioinformatics tertiary research analysis process in the form of a hierarchical task tree. To elicit the model, we integrated the trees resulting by the participants’ interviews in a unique structure. Some conflicts were present in their descriptions. In those cases, we opted for the solution adopted by the majority of the participants. When the same number of participants were supporting the contrasting opinions, we asked an expert bioinformatician who had not been interviewed to resolve the conflict, providing his perspective.

This representation is functional for several reasons. First, the tree presents the description of the process at many levels of abstraction, providing the right granularity for the specific problem. For this reason, the same model can be used to describe systems that work at different levels. At the same time, the tree embeds the *part of* relationship between parent nodes and children, providing the requirements necessary for the elicitation of all the operations that a tool must provide. Indeed, a tool developed for task *A* must support all the operations described by *A*’s children nodes. Even if there are more powerful tools to describe tasks, such as ConcurTask Trees [28], we decided to adopt hierarchical task trees because they are the most similar representation of the users’ responses we collected in the interviews. In addition, this is not the final model, but only a transitional representation before the adoption of the ontology formalism, as described later in this document.

**Fig. 3:**
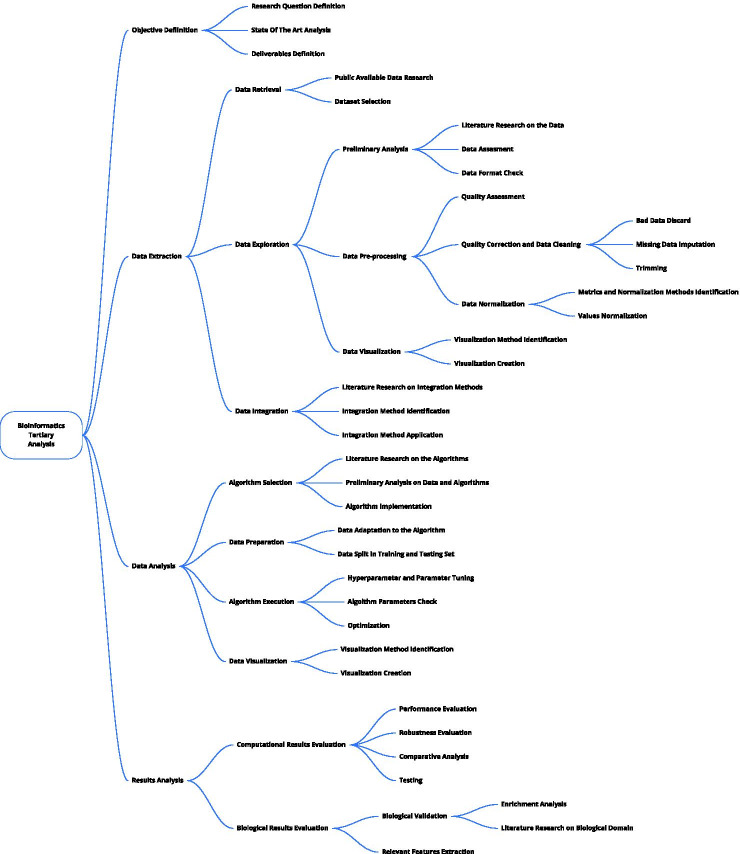
Hierarchical task tree retrieved from the empirical study and the literature research. It represents the typical pipeline of a bioinformatics research using different abstraction levels. Particularly, it is composed of four main-phases: *Objective Definition, Data Extraction, Data Analysis and Result Analysis.* Each of these is composed of many children that allows to better specify and clarify the steps that are usually computed

The resulting tree is shown in Fig. 3. All the people agreed that the tertiary analysis process could be divided into four main phases, typical in most data science applications: *Objective Definition, Data Extraction, Data Analysis,* and *Results Analysis*. Domain-specific distinctive traits emerge while going in-depth in the structure, that is, looking at the process at a finer granularity.

Participants agreed that the bioinformatics research process starts from the *Objective Definition*, i.e., the delineation of what a researcher want to find and obtain from that analysis. Three sub-tasks compose this task: *Research Question Definition, State of the Art Analysis,* and *Deliverables Definition*, corresponding to the definition of the question the researcher wants to answer, an analysis of the related works to that question and a definition of the results the researcher would like to show at the end of the pipeline. The last phase is usually performed together with the domain experts that will evaluate the results from the biological perspective. The output of the deliverable definition is a list of tables, plots, or a set of data necessary to verify the research hypothesis elicited in the *Research Question Definition* phase.

Once the purpose of the research has been defined, *Data Extraction* process begins. Also this task is divided in three parts: *Data Retrieval, Data Exploration,* and *Data Integration*. *Data Retrieval* begins with the *Research of Publicly Available Datasets*, in order to understand which, among the available data, can be used to answer the research question. This phase is concluded with the *Dataset Selection*. *Data Exploration* is the first phase in which the scientist gets in touch with the selected data. At first, a *Preliminary Analysis* is carried out for a first understanding of the data. This analysis is performed through a *Literature Research on the Data*, a *Data Assessment* phase, and finally a *Format Check*. Then, *Data Pre-processing* is performed to try to remove the noise from data. An initial phase of *Quality Assessment* is then carried out to understand noisy data. At that point, the *Quality Correction and Data Cleaning* process starts. It consists of three steps: *Bad Data Discard, Missing Data Imputation*, and *Trimming* to exclude extreme values and/or outliers. The pre-processing phase terminates with the *Data Normalization*, divided into *Metrics and Normalization Methods Identification* and the *Values Normalization*. During the exploration of the dataset, *Data Visualization* is essential to understand the nature of the data in the analysis. This phase is divided in two parts: *Visualization Method Identification* and *Visualization Creation*. The extraction phases concludes with *Data Integration*. In this phase, heterogeneous data are integrated to have a unique dataset on which to perform the analysis. Integration is the result of three subsequent processes: a *Literature Research on Integration Methods*, the *Integration Method Identification*, and finally the *Integration Method Application*.

Then, *Data Analysis* begins. This is the core of the project, data are analyzed through statistical and computational algorithms to extract information from them. Three phases compose the analysis: *Algorithm Selection, Data Preparation*, and *Algorithm Execution*. During *Algorithm Selection*, the most suitable algorithm is chosen. This process is supported by a *Literature Research on the Algorithm* to understand which is the current state of the art in similar works, *Preliminary Analysis on Data and Algorithms* to access the compatibility of the dataset and the selected algorithm(s), and finally the *Algorithm Implementation*. The *Data Preparation* phase is necessary to transform the dataset to be able to run the selected algorithm on it. To do that, first, there is the *Data Adaptation to the Algorithm*, followed by the *Data Split in Training and Testing Set*. The last step is fundamental to be able to evaluate the trained algorithms correctly. Finally, there is the *Algorithm Execution*. The operations performed in these phases vary a lot according to the algorithm. Accordingly, they can be grouped into three processes: *Hyper Parameter and Parameter Tuning, Algorithm Parameters Check*, and *Optimization*.

The fourth and last phase of the bioinformatics tertiary research process is *Results Analysis*. Here, the information extracted through the algorithms is converted into knowledge. To do that, first a *Computational Results Evaluation* is necessary to see if they are significant and therefore if they can be considered valid. The computational validation consists of *Performance Evaluation, Robustness Evaluation, Comparative Analysis*, and *Testing*. Then, *Biological Results Evaluation* is done to understand if the results can find an explanation from a biological perspective. Biological Results Evaluation comprises three tasks: *Biological Validation*, that is divided into *Enrichment Analysis* and *Literature Research on Biological Domain*, *Relevant Features Extraction*, and *Functional Genomic Analysis*.

### Validation

We run a literature-based analysis to validate the Hierarchical Task tree (Fig. 3). In particular, we want to assess its descriptive capabilities, and understand which are the properties of the research works that this model highlights.

**Fig. 4:**
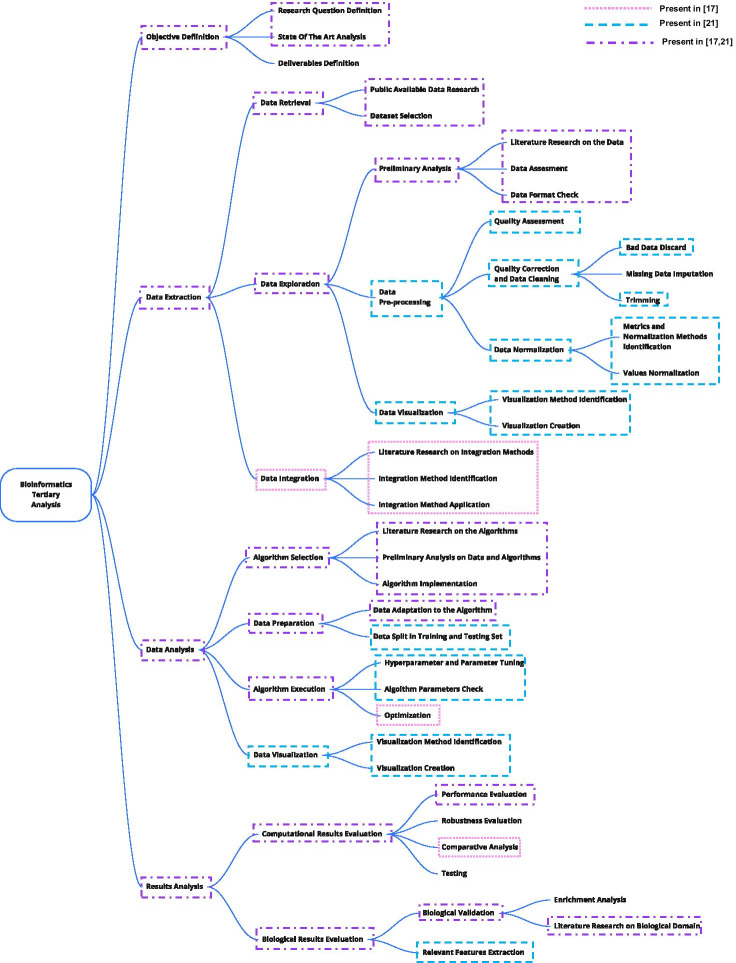
Example of the validation process on papers [46, 47]. The dotted pink rectangles are the tasks mentioned only by [47]; the slashed blue lines the ones mentioned only by [46]; the purple mixed lines the ones mentioned in both the papers. “Appendix” describes the mapping in details

We selected systematically 30 research and methodology papers in the field of bioinformatics tertiary analysis from two sources: the works from Genomic Computing Group (http://www.bioinformatics.deib.polimi.it/geco), given the direct contact with the authors in case of need for clarifications, and the most recent work published on *BMC Bioinformatics* journal. Papers were selected considering the title and the abstract. Particularly, we considered the most recent methodology or research articles dealing with tertiary bioinformatics analysis. We excluded all the software articles, in addition to those, we also excluded all the papers that did not use secondary analysis results as a starting point. Then, the papers were read extensively and mapped into the 36 leaves of the tree. A task had to be explicitly described to be considered in the paper. Two examples of this process are described in “Appendix” and represented in Fig. 4. Tables 1 and 2 show the results.

**Table 1:** Mapping between the leaves of the hierarchical task tree and the analyzed papers

	Tree Leaf	[46]	[47]	[48]	[49]	[50]	[40]	[39]	[51]	[52]	[53]	[54]	[55]	[56]	[57]	[58]
1	Research Question Definition	X	X	X	X	X	X	X	X	X	X	X	X	X	X	X
2	State of the Art Analysis	X	X	X	X	X	X	X	X	X	X	X	X	X	X	X
3	Deliverable Definition															
4	Public Available Data Research	X	X	X	X	X	X	X	X	X	X	X	X	X	X	X
5	Dataset Selection	X	X	X	X	X	X	X	X	X	X	X	X	X	X	X
6	Literature Research on the Data	X	X	X	X	X	X	X	X	X	X	X	X	X	X	X
7	Data Assessment	X	X	X	X	X	X	X	X	X	X	X	X	X	X	X
8	Data Format Check	X	X	X	X	X	X	X	X	X	X	X	X	X	X	X
9	Quality Assessment	X		X	X			X								X
10	Bad Data Discard	X		X	X	X		X	X		X			X		
11	Missing Data Imputation			X				X								
12	Trimming	X		X	X	X			X				X	X		
13	Metrics and Normalization Methods Identification	X			X				X		X					
14	Values Normalization	X			X				X		X					
15	Visualization Method Identification	X			X		X		X			X	X			
16	Visualization Creation	X			X		X		X			X	X			
17	Literature Research on Integration Methods		X						X	X						
18	Integration Method Identification		X						X	X						
19	Integration Method Application		X						X	X						
20	Literature Research on the Algorithms	X	X	X	X	X	X	X	X	X	X	X	X	X	X	X
21	Preliminary Analysis on Data and Algorithms	X	X	X	X	X	X	X	X	X	X	X	X	X	X	X
22	Algorithm Implementation	X	X	X	X	X	X	X	X	X	X	X	X	X	X	X
23	Data Adaptation to the Algorithm	X	X	X	X	X	X	X	X				X	X	X	X
24	Data Split in Training and Testing Set	X		X			X					X	X	X	X	X
25	Hyperparameter and Parameter Tuning	X		X			X	X			X	X		X	X	
26	Algorithm Parameters Check	X		X		X	X	X			X	X		X	X	
27	Optimization		X	X		X										
28	Visualization Method Identification	X			X	X		X	X		X		X	X	X	X
29	Visualization Creation	X			X	X		X	X		X		X	X	X	X
30	Performance Evaluation	X	X	X	X	X	X			X	X	X	X	X	X	X
31	Robustness Evaluation				X			X								
32	Comparative Analysis		X	X			X	X	X	X	X					
33	Testing						X					X	X			
34	Enrichment Analysis			X	X	X			X							
35	Literature Research on Biological Domain	X	X	X	X			X	X							
36	Relevant Feature Extraction	X								X	X					

Crossed boxes indicate the tasks described explicitly in the paper

**Table 2:** Mapping between the leaves of the hierarchical task tree and the analyzed papers

	Tree Leaf	[59]	[60]	[61]	[62]	[63]	[64]	[65]	[37]	[66]	[67]	[68]	[69]	[38]	[70]	[71]
1	Reseach Question Definition	X	X	X	X	X	X	X	X	X	X	X	X	X	X	X
2	State of the Art Analysis	X	X	X	X	X	X	X	X	X	X	X	X	X	X	X
3	Deliverable Definition															
4	Public Available Data Research	X	X	X	X	X	X	X		X	X	X	X	X	X	X
5	Dataset Selection	X	X	X	X	X	X	X	X	X	X	X	X	X	X	X
6	Literature Research on the Data	X	X	X	X	X	X	X	X	X	X	X	X	X	X	X
7	Data Assessment	X	X	X	X	X	X	X	X	X	X	X	X	X	X	X
8	Data Format Check	X	X	X	X	X	X	X	X	X	X	X	X	X	X	X
9	Quality Assessment	X									X				X	
10	Bad Data Discard							X			X					X
11	Missing Data Imputation															
12	Trimming				X						X	X				X
13	Metrics and Normalization Methods Identification	X							X			X		X	X	X
14	Values Normalization	X							X			X		X	X	X
15	Visualization Method Identification							X			X	X		X	X	X
16	Visualization Creation							X			X	X		X	X	X
17	Literature Research on Integration Methods						X				X			X	X	
18	Integration Method Identification	X					X				X			X	X	
19	Integration Method Application	X					X				X			X	X	
20	Literature Research on the Algorithms	X	X	X	X	X	X	X	X	X	X	X	X	X	X	X
21	Preliminary Analysis on Data and Algorithms	X	X	X	X	X	X	X	X	X	X	X	X	X	X	X
22	Algorithm Implementation	X	X	X	X	X	X	X	X	X	X	X	X	X	X	X
23	Data Adaptation to the Algorithm	X	X	X	X	X	X	X	X	X	X	X	X	X	X	X
24	Data Split in Training and Testing Set	X	X			X		X	X					X	X	X
25	Hyperparameter and Parameter Tuning		X						X						X	
26	Algorithm Parameters Check		X						X						X	
27	Optimization				X					X	X		X			X
28	Visualization Method Identification	X		X	X	X	X	X	X	X	X	X	X	X	X	X
29	Visualization Creation	X		X	X	X	X	X	X	X	X	X	X	X	X	X
30	Performance Evaluation	X	X	X	X		X	X		X	X	X	X	X	X	X
31	Robustness Evaluation												X			X
32	Comparative Analysis		X						X				X		X	X
33	Testing															
34	Enrichment Analysis	X							X					X		X
35	Literature Research on Biological Domain	X							X	X	X			X		
36	Relevant Feature Extraction								X							

Crossed boxes indicate the tasks described explicitly in the paper

**Fig. 5:**
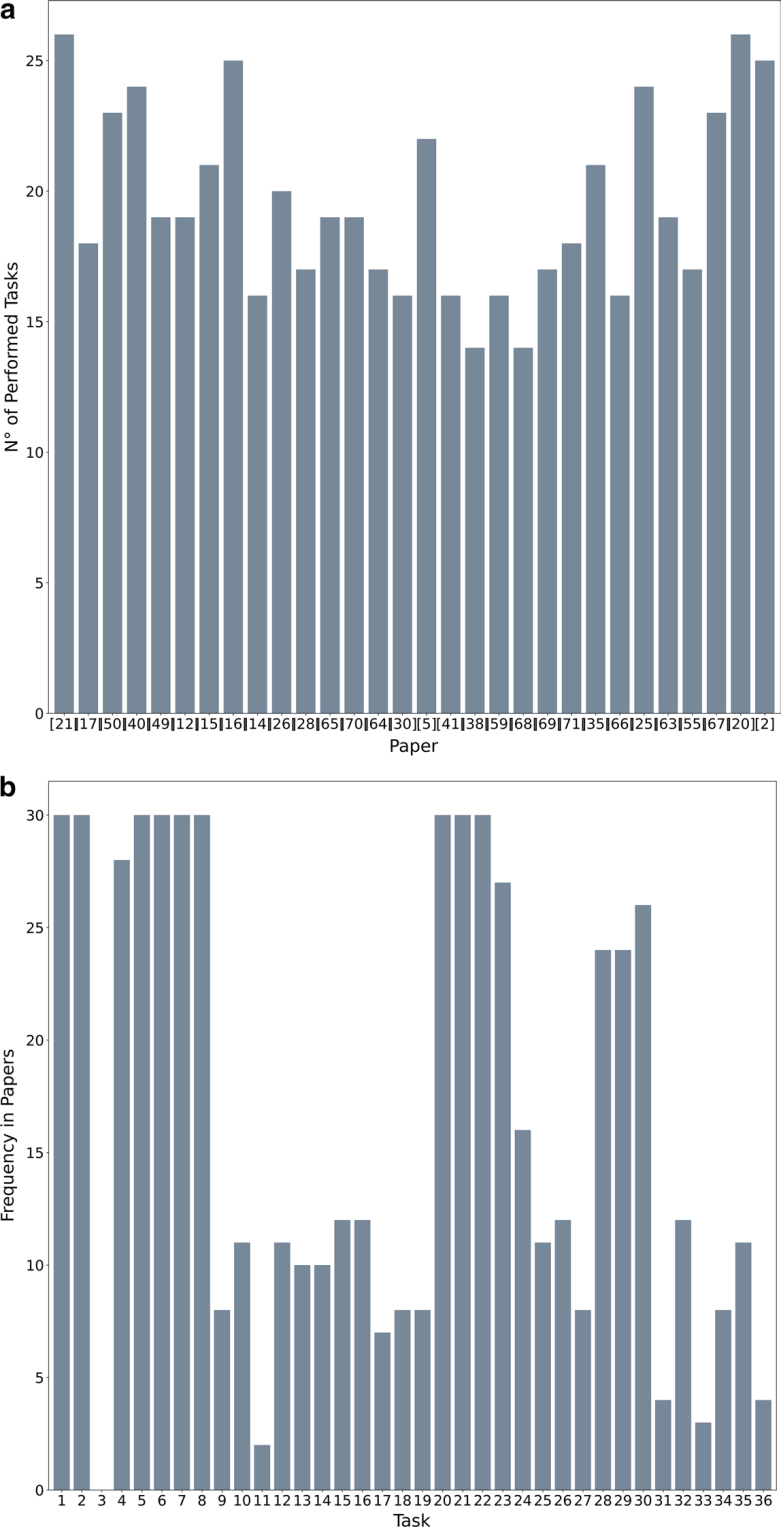
Barplots representing the validation step. **a** Represents the number of tasks present in each paper, i.e., the sum of the columns in Tables 1 and 2, **b** represents the number of papers that contains each task, i.e., the sum of the rows in Tables 1 and 2

Figure 5 shows the aggregates values, i.e., the frequencies of presence between tasks and papers. We were able to map every operation described in the paper to a task in the tree, proving the completeness of the model. On average, the number of tasks mentioned is around half of the 36 tasks, more precisely the average is 18.9. Whereas, the standard deviation is 3.0. The low standard deviation shows how the number of tasks is homogeneous in the description.

The same reasoning does not apply to the tasks: the distribution of number of mentions of the tasks is highly irregular. Indeed, the average is lower, i.e. 16.4, while the standard deviation is higher, i.e. 10.3. 10 tasks are present in all the papers analyzed (*Research Question Definition*, *State of the Art Analysis*, *Dataset Selection*, *Literature Research on the Data*, *Data Assessment*, and *Data Format Check*, *Literature Research on the Algorithms*, *Preliminary Analysis on Data and Algorithms*, *Algorithm Implementation* and *Data Adaptation to the Algorithm*), highlighting their importance in the description of the tertiary bioinformatics process. Among the most mentioned tasks there are *Public Available Data Research* (28/30) and *Performance Evaluation* (26/30). These steps are extremely important as well; the only two papers that do not consider public data are based on the dataset from a previous work [37, 38], whereas the other manuscripts that do not mention the performance evaluation validate their results with other techniques such as a *Comparative Analysis* [39, 40] or *Robustness Evaluation* [37, 39].

Not all tasks share the same popularity: five tasks are mentioned tree times or less (*Missing Data Imputation, Robustness Evaluation, Testing*). In particular, the *Deliverable Definition* is never described in the papers considered. The reason is that even if this phase is crucial for the success of a research project, often it is implicit and not clearly stated. Also in the interview process it was subtended most of the times (7/8). The only researcher who mentioned it explained that this step is crucial when the results are validated by non-computer scientists.

Looking at how the tasks appears, only 36 tasks out of 503 appear isolated, i.e., without the adjacent tasks being described. Moreover, the isolated tasks are not equally distributed, but all belong to *Quality correction and Data Cleaning* and *Results Analysis* sub-tasks. The motivation lies in the different nature of the tasks: quality correction and data analysis operations are independent and not consecutive steps of a unique process. In results analysis this tendency is even stronger; indeed, only a few papers apply more types of validation in the same branch of the tree.

A more attentive analysis shows that some adjacent rows (i.e., tasks) appear always coupled: *Visualization Method Identification* and *Visualization Creation*, *Metrics and Normalization Methods Identification* and *Values Normalization*, and the triple *Literature Research on Integration Methods*, *Integration Method Identification*, *Integration Method Application*. Also in this case the semantics of the operations justifies this behavior: the first task of the couples is the preliminary and necessary task for the correct execution of the second one, like in the case of understanding how to visualize data before plotting them.

Lastly, an interesting relationship emerges between *Data Pre-processing* operations and *Biological Results Evaluation*. The number of sub-tasks mentioned by the papers in the two branches is strongly correlated: the Pearson correlation value between the two values is 0.8417 with a *p*-value of $5.6166{\text{e}}{-09}$. This relationship is justified by the nature of the papers. Indeed, the interest in pre-processing the data is typical in works that want to draw biological conclusions on those data. From this consideration we can identify two main categories of works: the computational contributions that focus the research on a new algorithmic solution for tertiary analysis, and biological ones, that aim at finding not only computational methods but also new biological advancements from the biological perspective. We find that the number of tasks in *Data Pre-processing* and in *Biological Results Evaluation* is a good indicator of the category in which the manuscript falls: if the manuscript has only a computational scope, we can notice that the *Data Pre-processing* steps are almost always skipped, otherwise, at least one of them is almost always performed and the *Biological Results Evaluation* is present.

## Results

Even if the Hierarchical Task Analysis produced a detailed description of the Bioinformatics Tertiary Research Process, this model had some limitations. The *part of* relation was not sufficient to describe the process adequately in detail. The tree-based description does not provide any information on the output of the tasks. On top of that, the hierarchical structure is not flexible enough to describe the precedence among tasks. In fact, for most siblings, the precedence order is strictly given by order of appearance in the tree. However, in some part of the process, there is not such a strict precedence order, like in the case of *Literature Research on the Data* and its siblings, making a depth-first visit algorithm not sufficient to determine the task precedence. Finally, the hierarchical task tree is not declared with the typical declarative languages used for ontologies, preventing it from being integrated and used with other models and from using tools for their exploitation and analysis.

**Fig. 6:**
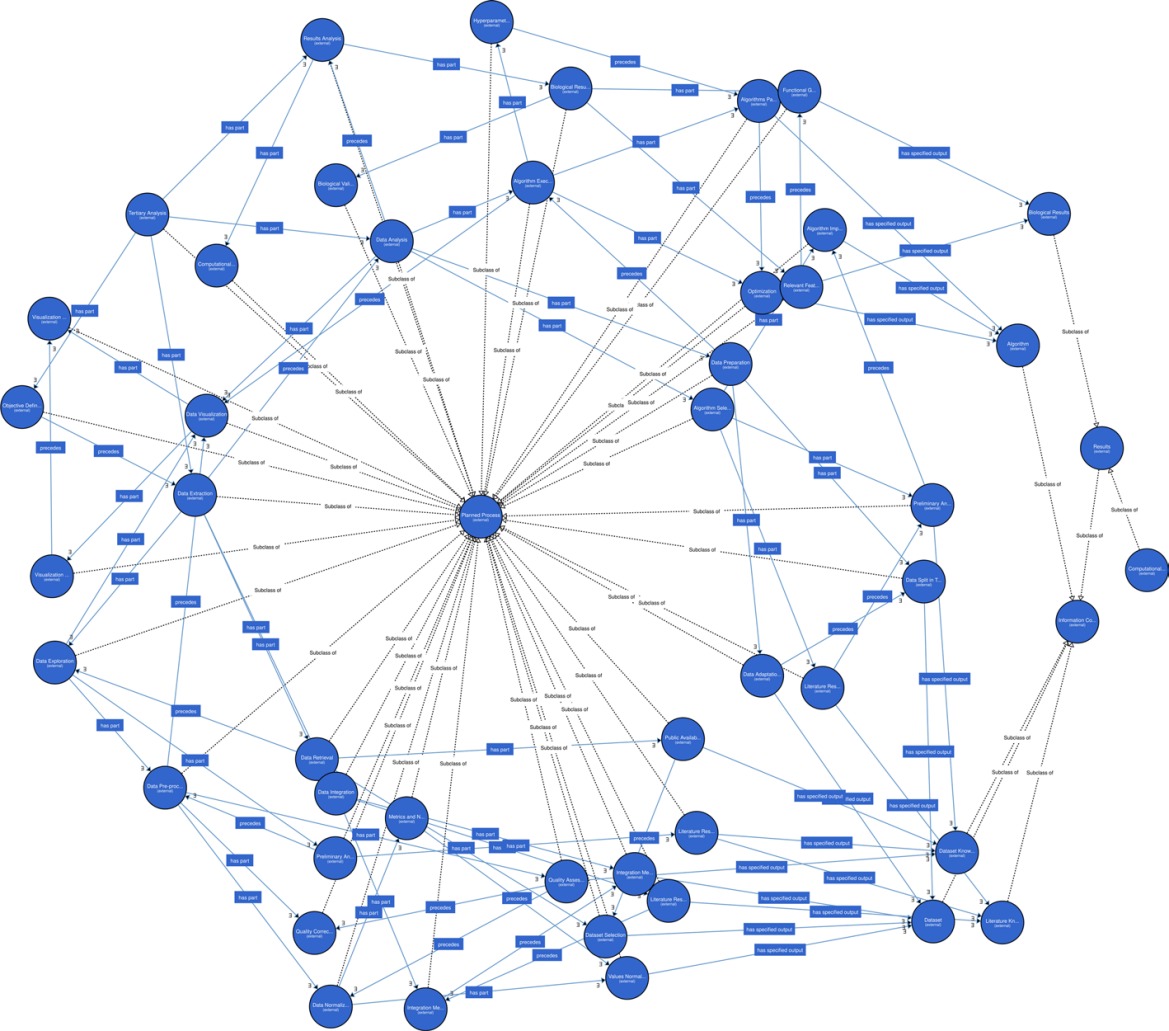
**a** Graphical representation of the model. Particularly, it represents the relations that exist among the different classes. To interactively explore the model, the reader can upload the file .owl n the supplementary material on an ontology visualization tool, such as http://www.visualdataweb.de/webvowl. **b** Graphical representation of a section of the model. Particularly, it represents the path from the Tertiary Analysis to Data Pre-processing with all its leaves nodes

To translate the model we used Web-protege [41], a web-based graphical user interface to model OWL-based ontologies. A graphical representation of the results and a detail are shown in Fig. 6. The most recent version of the model is available at this link: https://github.com/peempi/btap

For these reasons, we evolved our model adopting the formalism of OWL2 Web Ontology Language [42]. We chose OWL over OBO [43] format since it provides wider support on the semantics. Our model inherits the Resource Description Framework (RDF) [44] representation of data, according to which the model is coded through triples representing subject-predicate-object. This representation implicitly creates a directed graph of the ontology, as shown in the Fig. 6. Finally, OWL builds its language on the RDF Schema [45], providing an expressive manner to describe the elements in the ontology and their relations. The result is a decidable fragment of first-order logic. In other words, we can build OWL reasoners that can answer questions on the model in a finite time and number of steps.

The resulting representation counts 70 classes and 3 relations. The upper level consists of OBI classes *Information Content Entity* and *Planned Process*. Planned processes are processes performed typically by a researcher and produce an output in the form of an Information Content Entity. Information Content Entities represent knowledge, data, and results created by the successful execution of a planned process. Note that a successful execution does not imply a positive outcome. We refer to processes as successful when their procedure is executed correctly, and some output is produced, no matter if it confirms o denies the research hypothesis. Among Information Content Entities, there is the *Results* subclass that is the parent of all the Information Entities that produce new knowledge on the research topic.

Classes are related by the three relations:

- *Has Part* relation defines which sub-processes define a process. This relation is equivalent to the parent-children relations in the hierarchical task tree. Differently from the tree representation, a sub-process common to more processes can be the object of multiple relations, removing the duplicates that the tree suffered from (e.g., Data Visualization). Any process can have zero or more *Has Part*.
- *Has Specified Output* describes the output for a process. For this reason, in such a relation, the subject must be a Planned Process, whereas the object is an Information Content Entity. A Planned Process can be the subject of zero or more Has Specified Output, and an Information Content Entity can be the object of one or many Has Specified Output Planned Processes. The following property holds: given *A* and **B** Planned Processes objects and *C* being an Information Content Entity, if *(A has part B)* and *(B has specified output C)*, then *(A has specified output C)*. These inferred properties are not explicitly reported in the ontology.
- *Precedes* relation express the temporal constraint in the process. Both the subject and the object must be Planned Processes. Intuitively, if *A* and *B* are Planned Process and *(A precedes B)* holds, then the output of *A* is necessary for the correct execution of *B*. Precedes relation is transitive: if *A, B*, and *C* are planned processes, if *A precedes B)* and *(B precedes C)*, then *A precedes C*. Finally, the Precedes relation is specified explicitly only between siblings. The following property holds: if *A, AA, B*, and *BB* are planned processes, *(A has part AA)*, *(B has part BB)*, and *(A precedes B)*, then *(AA precedes B)*.
- *Requires* relation models the necessity of preliminary operations for the execution of the task. Intuitively, if *A* and *B* are Planned Processes and *(B requires A)*, if an instance of process presents *B*, then it must present *A* as well.

## Discussion

This representation has many potential uses and applications. The first and more immediate one is the possibility of using a unified terminology to describe terminology, as we already showed while validating the hierarchical task tree through literature works. Bioinformaticians can use the model presented as a reference to be guided through the process, having a detailed step-by-step guide of all the procedure. In the same way, work groups can coordinate the project, having a schematic representation of the research pipeline to use as a reference. Finally, software engineers can exploit the model to design new tools to support bioinformatics, these tools are more usable and predisposed for the integration in the research pipeline. The formalism adopted makes the process model machine readable: not only tools can be modelled on it, but they can exploit the representation to embed the knowledge of the process and use it as a base for creating the operation pipeline, or to check on the operations requested from the users.

### Using the model to design new tools

We can now provide an example of how the model can be used as a reference for the design of new bioinformatics tools. We will exemplify the procedure by designing a Conversational Agent that guides bioinformaticians and biologists in extracting genomic data from a database according to the process in the hierarchical task tree. The task we want to illustrate corresponds to the *Data Extraction*.

The development of a tool requires a profound knowledge of the task to be supported. In particular, a Conversational Agent requires a process on which to base the dialogue. Thus, the ontology-based representation helps in the definition of this flow. The ontology-based representation immediately provides the sub-tasks to be supported by the application: *Data Retrieval, Data Exploration,* and, if necessary, *Data Integration*. Consequently, such a dialogic interface must support three main moments that are mapped into three tasks. Reasoning in the same way, we can iteratively define the requirements of the conversation until we reach the tasks that do not include any sub-tasks. This means that, for example, for the *Data Retrieval* phase, the CA must support a conversation that keeps into consideration two sub-tasks, i.e., *Public Available Data Research* and *Dataset Selection*. These two tasks are leaves of the hierarchical task tree, thus, they are not expanded further. The conversation is built upon these leaves nodes. The outcome of this initial phase is a precise description of all the moments that the conversation must support according to the step in the hierarchical task tree.

Thanks to the ontology-based representation of the bioinformatics tertiary process, we were able to define the main steps the interaction has to touch during the process. The next step is to design the conversation: for each sub-task, we can define a portion of the dialogue to guide the user accomplishing that step. In parallel, we will define the back-end operation required to handle the user input and elaborate the data.

The result is a Conversational Agent that guides the user step by step in the data extraction. The session will start by asking which datasets to select from the publicly available data. Then, users can refine their research by applying filtering operations to improve the research results. At this point, the Retrieval phase is complete, and the agent proceeds to the exploration task by showing the users the meaning of the selected data and their format. The conversation must now guide researchers into the quality assessment, data cleaning, and normalization of the data. At every step, the agent will illustrate the possible options, for example, by asking the users whether they want to impute missing data or which metrics they want to use for normalizing the data. Graphs and visualization must be implemented to support the whole exploration phase. Finally, if the data come from multiple sources, the conversational agent will guide the researchers through the integration procedure.

The conversation will end with the download of the datasets to be used for the analysis. The ontology-based representation of the tertiary analysis allows obtaining a model for the process the CA has to follow. The model provides a basis on which the conversational agent translates users’ utterances into an operational workflow for the datasets creation. In addition, knowing the operation workflow, the system can proactively support researchers during the interaction, for example, by suggesting the most common operations or providing personalized recommendations based on the specific session.

Furthermore, with the ontology-based representation, it is easy to map inputs and outputs for each phase of the process. This possibility of dealing with the definition of input and output formats of the tool is another advantage of exploiting the model in the design phases. In fact, thinking of the new application not as a stand-alone product but as an element of a broader pipeline allows designers and developers to create a product that can be easily integrated within the research workflow. This means that the tool should accept as input the results of the former phase and produce something compatible with the main Data Analysis tools. In the case of this example, the *Data Extraction* task is positioned between *Objectives Definition* and *Data Analysis*. *Objective Definition* outputs are a *Research Question* and *Deliverable Requirements*; the conversational agents should be able to acquire this kind of information and suggest data accordingly. *Data Analysis* takes a dataset as input. Therefore the designed tools should produce a dataset in one of the most common data formats (e.g., .csv).

## Conclusions

In this paper, we presented the work that, starting from an empirical study, leads to the creation of a model to describe the bioinformatics tertiary research process. Our model is the result of a task analysis that originates from the expertise of bioinformaticians, gathered through interviews, and is validated through a systematic analysis of the works in the literature. Finally, we provide some examples of how the proposed model can be used in practice to analyze research processes and design new tools.

Our work fills a gap in the current literature, providing a reference to describe bioinformatics tertiary research process in a single framework. Our model is thought to unify the jargon in this discipline, to create a standard terminology to be used in bioinformatics research. Our model is not complete, and probably it will never be: bioinformatics is an arising discipline in continuous evolution. In the future, we aim at further expanding the process model and at integrating it to improve its soundness.

Differently from task modeling frameworks, such as ConcurTaskTree [28], the ontology-like formalism allows the representation not only of the temporal precedence of the tasks, but also of other kinds of information, such as the nature of the tasks, their outputs, and their similarities. As shown, the power of our ontology-based representation is not limited to its descriptive capabilities: such a formalism is an expressive framework that enables developers to design tools intended to be integrated within the research pipeline. Contrarily to major bioinformatics ontologies, our work models the bioinformatics tertiary analysis process: an emerging field that exploits the data gathered through procedures modeled in ontologies such as [32]. For this reason, our model is thought not to replace existing ontologies, but to complement them providing an initial formal description of the new frontiers of bioinformatics research: our ontology-like formalism allows the integration within the well-established ontologies in bioinformatics domain, as we aim to do in the future.

In this perspective, our research paves the way to a new era of bioinformatics, where tools are not only designed to be effective, but also integrated and easy-to-use, therefore allowing the domain experts (such as clinicians and biologists) to approach the full potentialities of the discipline.

### Supplementary Information

**Additional file 1.** Hierarchical task tree generated by Participant 1.

**Additional file 2.** Hierarchical task tree generated by Participant 2.

**Additional file 3.** Hierarchical task tree generated by Participant 3.

**Additional file 4.** Hierarchical task tree generated by Participants 4–5.

**Additional file 5.** Hierarchical task tree generated by Participant 6.

**Additional file 6.** Hierarchical task tree generated by Participant 7.

**Additional file 7.** Hierarchical task tree generated by Participant 8.

## Data Availability

Not applicable.

## Appendix: Examples of validation process

Here we provide two simple examples of the mapping between the tree and two different research articles and how they help us confirm and improve our predefined tree. Figure 4 presents the validation of the hierarchical task tree highlighting the mapping with the papers that follow.

In the study made by [46], the authors explain their research question, i.e., using deep approaches to model non-linear relationships to prove useful for breast cancer subtyping. The definition of the research question is the first leaf in our tree, as Fig. 3 shows. After the *State of the Art Analysis*, the second step in our hierarchical task tree, in which they analysed existing ways for analyzing and study BRCA (i.e., breast invasive carcinoma) biomarkers, they studied the available data. Particularly, as suggested by the Data Retrieval path, they selected data from TCGA and ARCHS4. They selected gene expressions, miRNA expressions, and CNA data. Following the paper, they modified and filtered the data according to what they wanted. This is the *Data Pre-processing* step. From reading the paper, it seems that they do not need any integration step. Thus, they continued their analysis with the *Algorithm Selection* and *Algorithm Implementation*. During the explanation of the methods, they referenced already used algorithms. This proves the performed *Literature Research on the Algorithm*. Furthermore, it comes out from the article that they split the data into training and test sets (*Data Split in Training and Testing Sets* is in Data Preparation branch) and that they applied *Hyper-Parameter Tuning* (as in *Algorithm Execution* branch). Finally, they explained the validation method used. Using the names in the hierarchical task tree, they evaluate the results using a *Performance Evaluation*. In addition to that, they applied an *Extraction of Relevant Features*. Thus, this study confirms many of the steps included in our tree. Particularly on 57 nodes of the tree, we could count at least 44 in the paper. It was clear from the analysis that some nodes were not needed, like all the nodes in the Data Integration branch. Whereas other nodes are not clearly stated from the paper, thus we could not assess if they were performed or not. However, even if not all tree nodes are in the research, all the steps present in the paper are also present in the tree. Moreover, this analysis helps us to confirm the *Algorithm Implementation* node that was in a doubt position due to the interviews. In addition to that, a first comparison shows that the order of the applied process in5 [46] is very similar to our hierarchical tree, giving another confirmation of the validity of the defined pipeline. As we expected, not all the nodes were present in the study, and it was not possible to define all the positions. Indeed, the order with which some tasks were computed was not as clear as in the tree.

The second example is taken from [47]. It is interesting because it shows complementary aspects with respect to the previous one. In the introduction, the research question the authors want to solve is clear, i.e. discover novel drug-protein target interactions, new drug annotations, and new drug-disease associations through an innovative method employing shortest paths in Non-negative Matrix Tri-Factorization (NMTF). After the *Research Question Definition*, the *State of the Art Analysis* is explained. It is not clear from the paper if they defined the deliverables or if this step was skipped. The next Section is regarding the algorithm, and only the next one explains the data used. However, even if in the paper the two branches Data Extraction and Data Analysis, are switched, it is clear that the description on the paper does not reflect the actual order in which operations have been executed. This is interesting since it highlights the differences between the real pipeline of bioinformatics research and how the pipeline is told in an article’s narrative. Thus, it strengthens the necessity of having a well-defined ontology to represent it. Going on in the paper, we can find the data extracted for the *Dataset Selection*, they used DrugBank, Reactome, Therapeutic Target Database, and BioGrid.

In the method Section, the authors explain how they structure the *Data Integration* protocol. The description of this task is of particular interest since it validates a section of the tree described by only two of the interviewed people. Then, the implementation of the algorithm is explained. Also, in this article *Literature Research on the Algorithm* is implied, but it can be retrieved from all the cited articles used to implement the method. Given the nature of the work, different from the previous paper, they do not adopt either *Data Split in Training and Testing Sets* or *Hyperparameter Tuning*. However, they directly pass to the *Algorithm Execution*, and they apply some *Optimization* on the algorithms.

For the *Computational Results Evaluation*, they applied the *Performance Evaluation* using Average Precision Score, False Positive Rate, and Area Under the Curve. To further validate the results, they also computed a *Comparative Analysis*, explained in Section “Computational Validation Methods and Prediction Performances”. For the *Biological Results Evaluation*, they use only *Literature Research on Biological Domain*.

As in the previous paper, some of the steps are not explicitly reported, and others are not clearly stated. However, we were able to match 33 nodes of the tree. Also this article proves the validity of the elicited tree: all the operations described in the document were classifiable in one node of the tree.

## Abbreviations

OWL: Web Ontology Language
GUI: Graphical User Interface
GOMS: Goals, Operators, Methods and Section rules
OBI: Ontology for Biomedical Investigations
BFO: Basic Formal Ontology
IAO: Information Artifact Ontology
OBO: Open Biological and Biomedical Ontology
RDF: Resource Description Framework
CA: Conversational Agent
