# Supplementary figures and images for: Modelling the bioinformatics tertiary analysis research process
Source: BMC Bioinformatics. 2021 Sep 30;22(Suppl 13):452. doi: 10.1186/s12859-021-04310-5 (PMC8482564; doi:10.1186/s12859-021-04310-5)

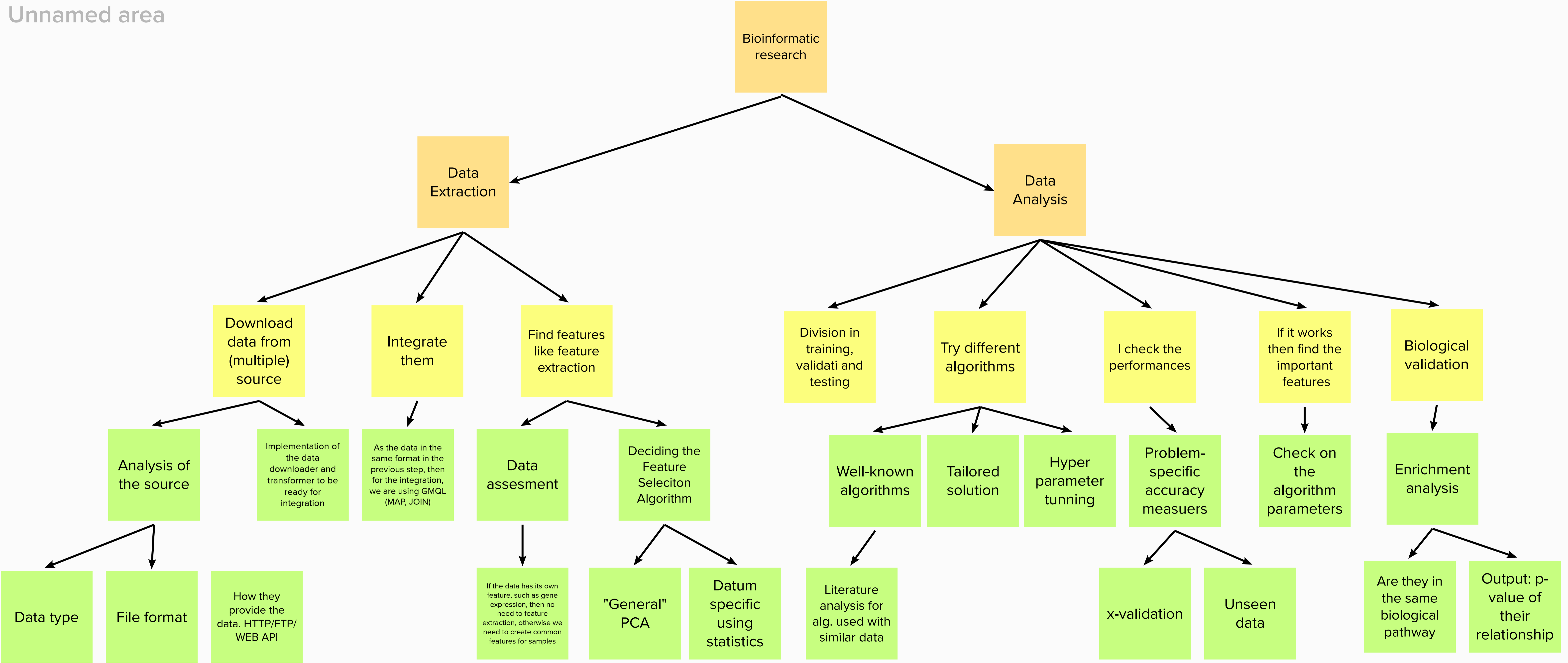

Supplement: Supplementary file 1 — Additional file 1. Hierarchical task tree generated by Participant 1. [file 12859_2021_4310_MOESM1_ESM.pdf]

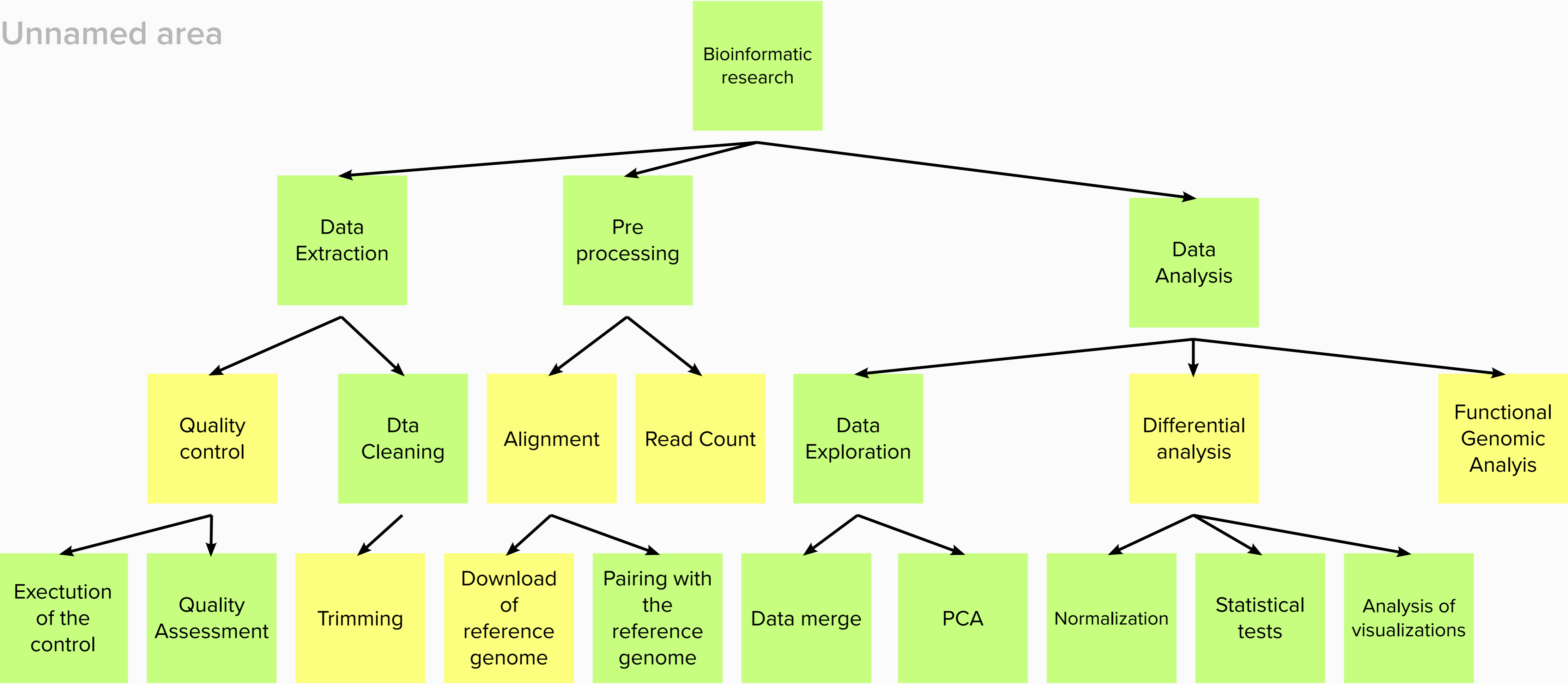

Supplement: Supplementary file 2 — Additional file 2. Hierarchical task tree generated by Participant 2. [file 12859_2021_4310_MOESM2_ESM.pdf]

1- Your process

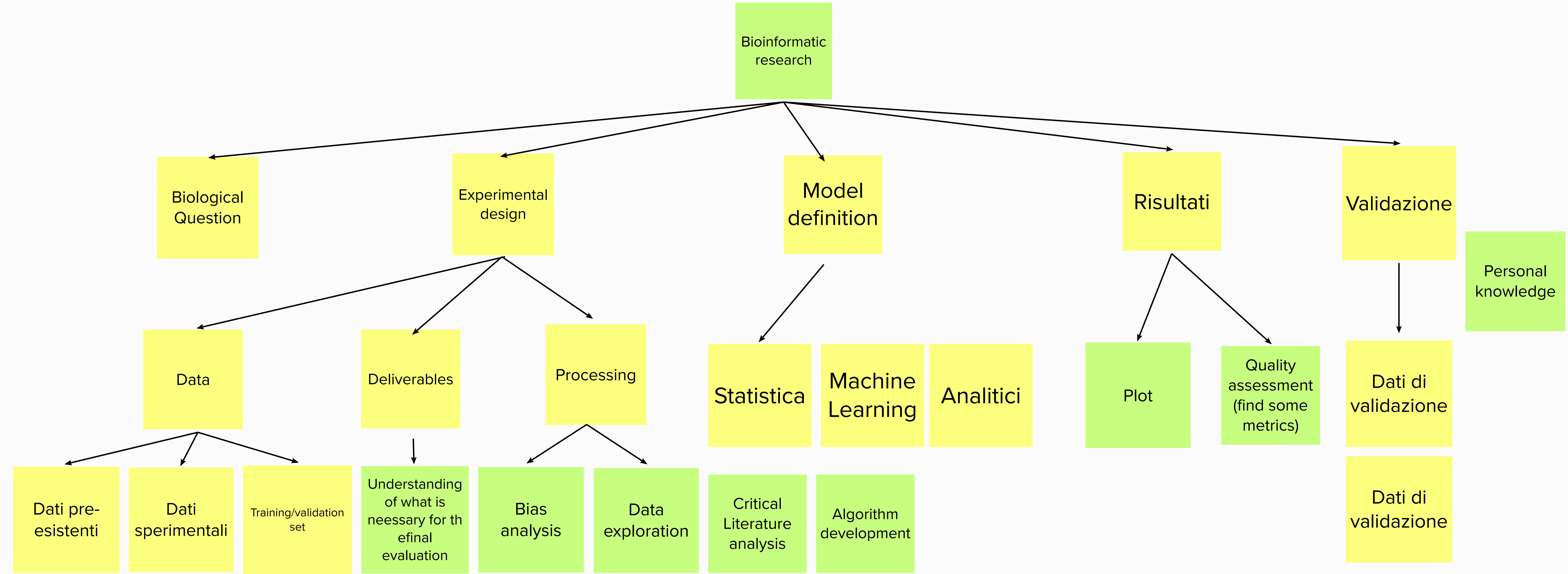

Supplement: Supplementary file 3 — Additional file 3. Hierarchical task tree generated by Participant 3. [file 12859_2021_4310_MOESM3_ESM.pdf]

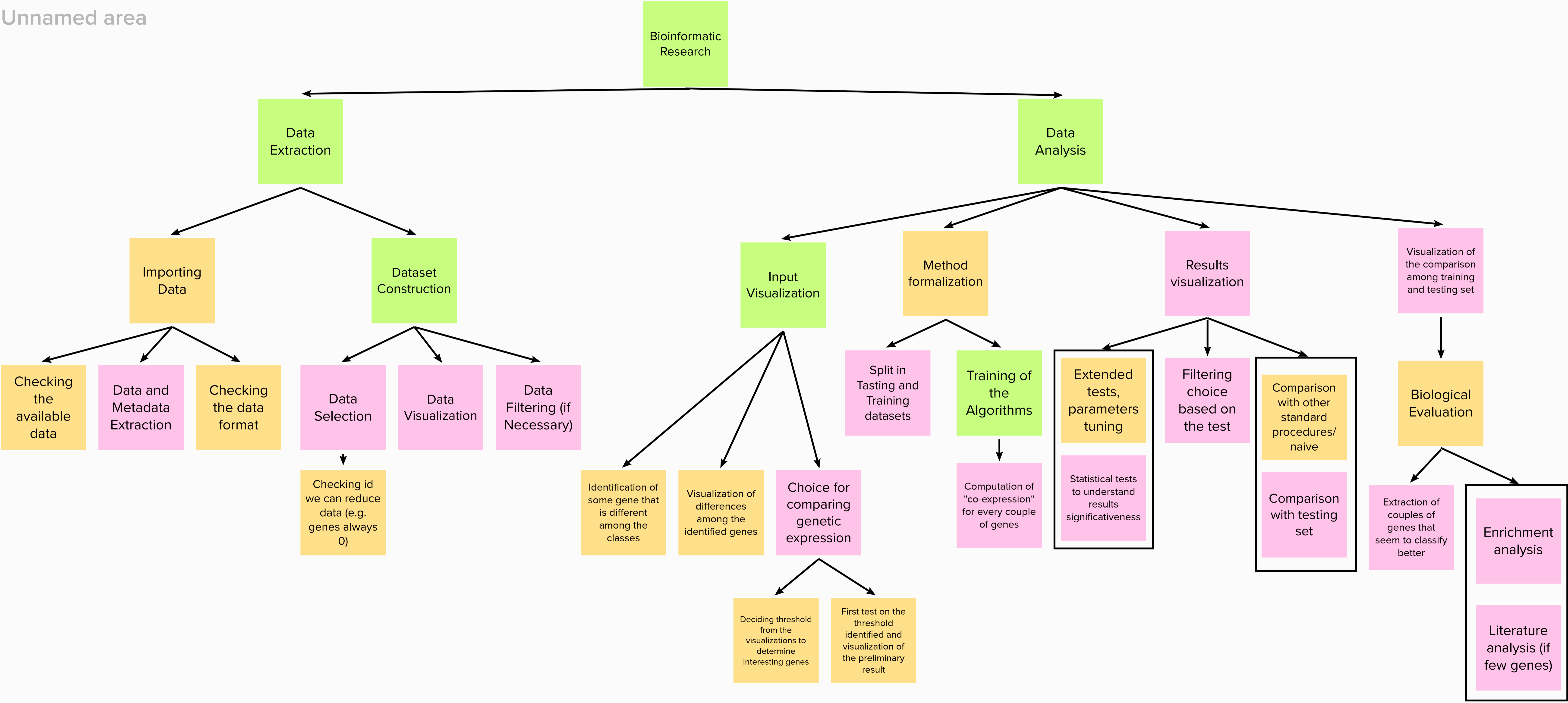

Supplement: Supplementary file 4 — Additional file 4. Hierarchical task tree generated by Participants 4–5. [file 12859_2021_4310_MOESM4_ESM.pdf]

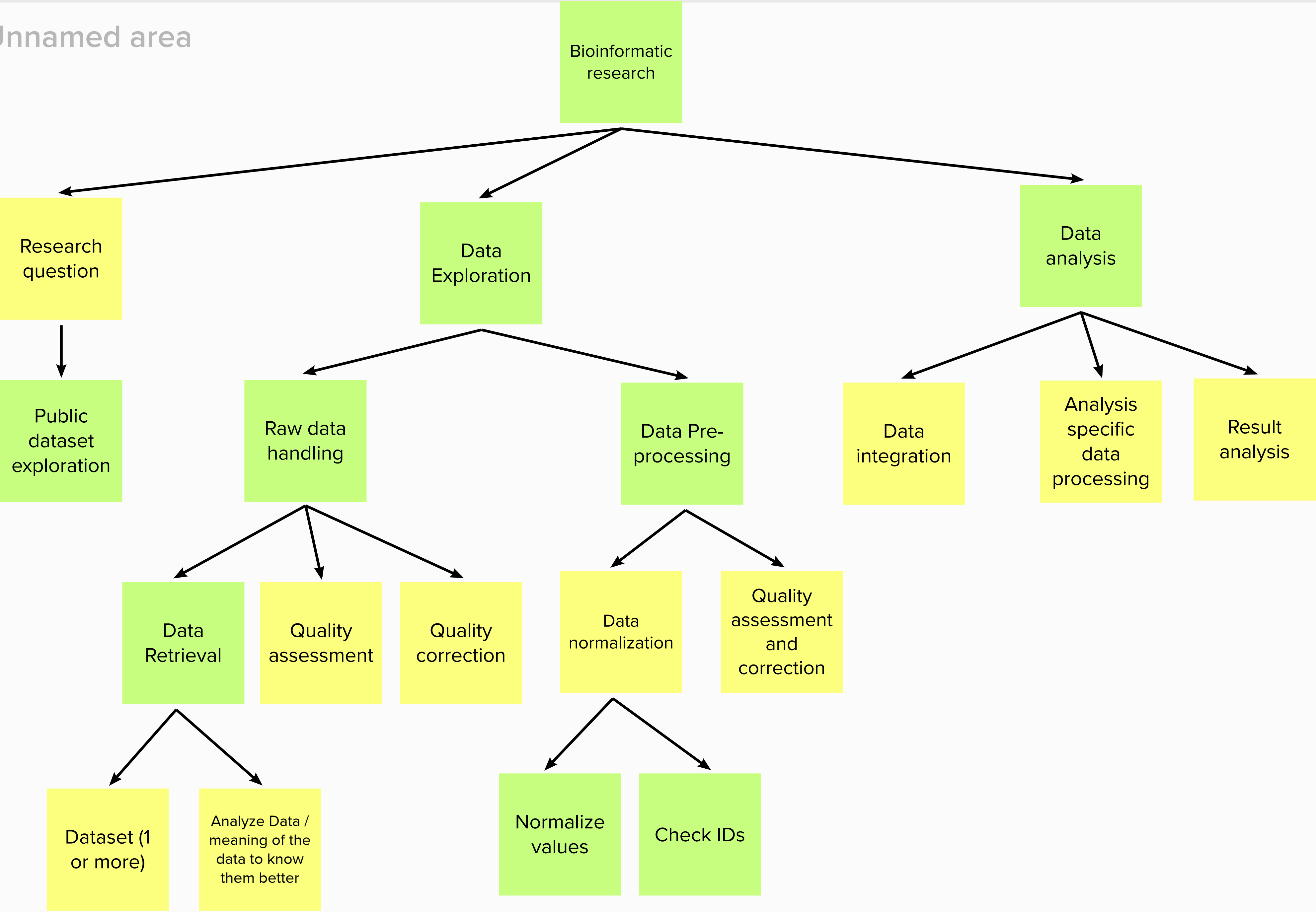

Supplement: Supplementary file 5 — Additional file 5. Hierarchical task tree generated by Participant 6. [file 12859_2021_4310_MOESM5_ESM.pdf]

Phase 3

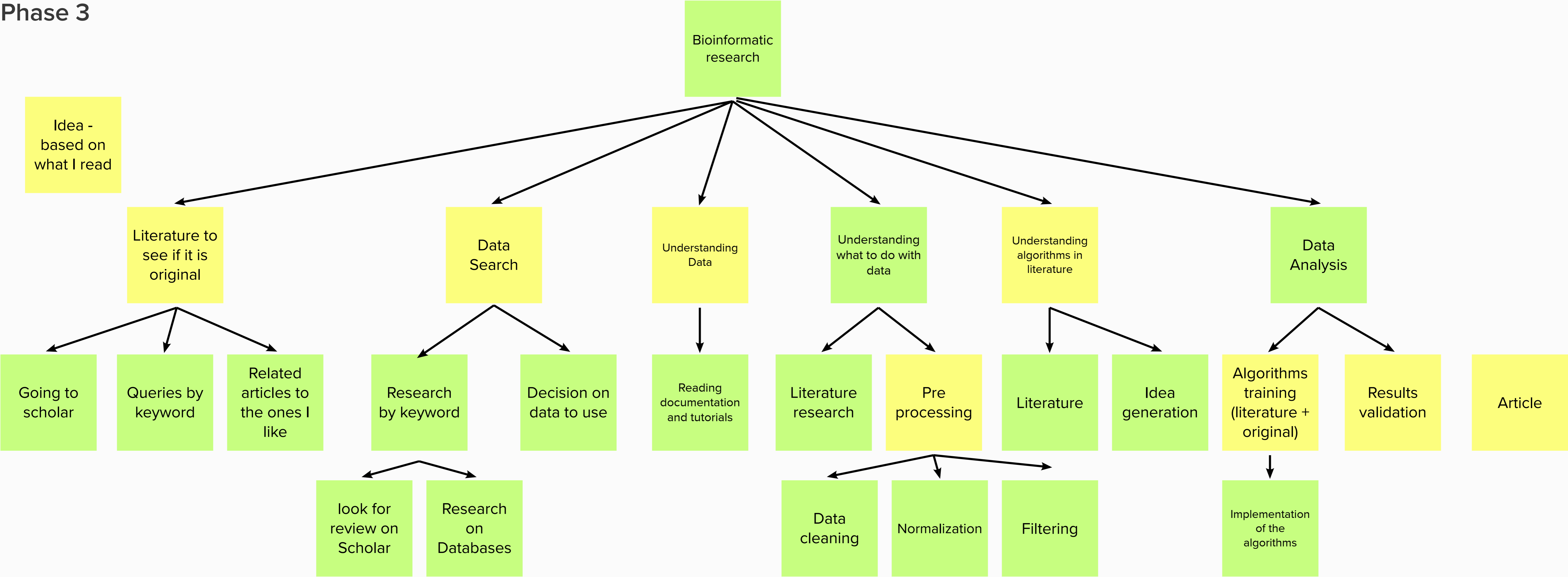

Supplement: Supplementary file 6 — Additional file 6. Hierarchical task tree generated by Participant 7. [file 12859_2021_4310_MOESM6_ESM.pdf]

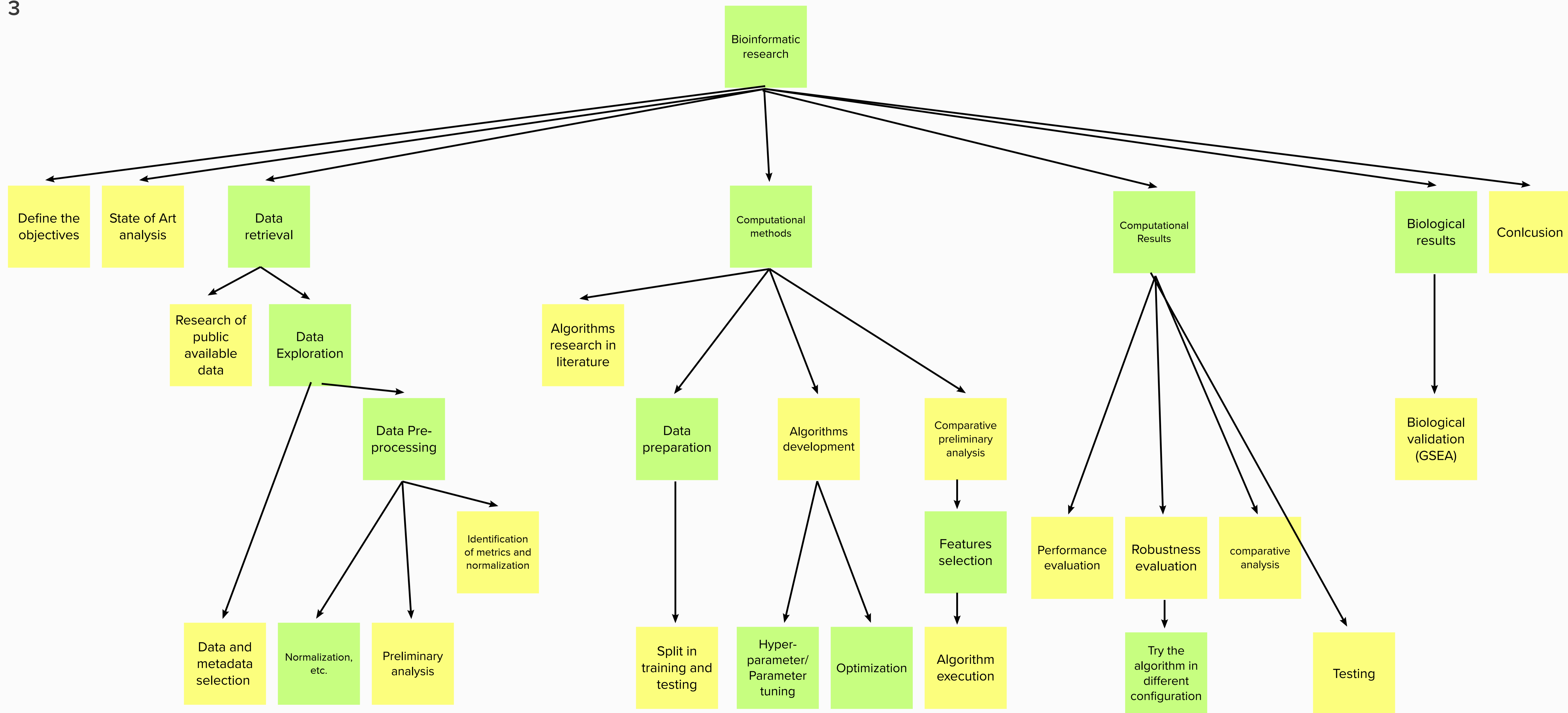

Supplement: Supplementary file 7 — Additional file 7. Hierarchical task tree generated by Participant 8. [file 12859_2021_4310_MOESM7_ESM.pdf]
